# Crosstalk Between Intestinal Microbiota Derived Metabolites and Tissues in Allogeneic Hematopoietic Cell Transplantation

**DOI:** 10.3389/fimmu.2021.703298

**Published:** 2021-08-27

**Authors:** Hideaki Fujiwara

**Affiliations:** Department of Hematology and Oncology, Okayama University Hospital, Okayama, Japan

**Keywords:** graft-versus-host disease, microbial metabolite, dysbiosis, microbiota, allogeneic stem cell transplantation

## Abstract

Allogeneic hematopoietic stem cell transplantation (allo-HSCT) is an evidence based- cellular immunotherapy for hematological malignancies. Immune reactions not only promote graft-versus-tumor effects that kill hematological malignant cells but also graft-versus-host disease (GVHD) that is the primary complication characterized by systemic organ damages consisting of T-cells and antigen presenting cells (APCs) activation. GVHD has long been recognized as an immunological reaction that requires an immunosuppressive treatment targeting immune cells. However immune suppression cannot always prevent GVHD or effectively treat it once it has developed. Recent studies using high-throughput sequencing technology investigated the impact of microbial flora on GVHD and provided profound insights of the mechanism of GVHD other than immune cells. Allo-HSCT affects the intestinal microbiota and microbiome-metabolome axis that can alter intestinal homeostasis and the severity of experimental GVHD. This axis can potentially be manipulated *via* dietary intervention or metabolites produced by intestinal bacteria affected post-allo-HSCT. In this review, we discuss the mechanism of experimental GVHD regulation by the complex microbial community-metabolites-host tissue axis. Furthermore, we summarize the major findings of microbiome-based immunotherapeutic approaches that protect tissues from experimental GVHD. Understanding the complex relationships between gut microbiota-metabolites-host tissues axis provides crucial insight into the pathogenesis of GVHD and advances the development of new therapeutic approaches.

## Introduction

Hematological malignancies, such as leukemia, lead to high mortality, especially in the elderly. Allogeneic hematopoietic stem cell transplantation (allo-HSCT) is an immune therapy that has become widely used for the treatment of life-threatening hematological malignancies and congenic immune deficiencies (1). However, many complications following allo-HSCT occur due to immune-related reactions, the most challenging and serious of which is graft-*versus*-host disease (GVHD) (2). GVHD is composed of complexes of immune cells and tissues (3). After T-cells activation and migration to GVHD target organs, such as the lung, gut, liver and skin, cytotoxic T-cells and helper T-cells attack target cells in these target organs. Once GVHD develops, recipients require additional immunosuppressive therapies using steroids in addition to GVHD preventive immunosuppressants consisting of calcineurin inhibitors. However, GVHD is frequently refractory to standard steroid therapy and has a dismal prognosis, with only 5–30% overall survival (4). In steroid-refractory GVHD, novel treatment approaches include the use of anti-thymocyte globulin, extracorporeal photopheresis, mesenchymal stromal cells, mycophenolate mofetil, everolimus, sirolimus, etanercept, infliximab, and ruxolitinib (5). Intensified immunosuppressive therapies for aggravated GVHD suppress immune reactions and permit bacterial translocation from injured intestinal epithelium cells; this leads to subsequent systemic infections that increase the release of pathogen-associated molecular patterns (PAMPs) and damage associated molecular patterns (DAMPs). This negative feedback loop can result in fatal GVHD. In addition, excessive immunosuppression increases malignancy relapse after HSCT. Thus, immunosuppressive therapies are limited.

Current focus has been shifted to the target tissues, the third player in GVHD, in addition to APCs and T-cells (3). The role of tissue-intrinsic factors that might contribute to the regulation of GVHD severity has been largely overlooked. Tissue-specific programs contribute to target tissue resilience, repair, and regeneration and mitigate the severity of GVHD without altering the load or function of alloreactive immune cells (6). In this context, the gastro-intestinal (GI) tract has close interactions with the microbiota, and recent genomic microbial analyses have revealed intricate connections between the microbiota and various diseases. Particularly, in GVHD, disturbance of microbial composition, that is, dysbiosis, is strongly associated with poor outcomes after allo-HSCT (7). However, whether a causal relationship exists remains unclear. In this review, we focus on the connection among gut microbiota, microbial metabolites and intestinal environment that affect GVHD. We also discuss the recent findings on microbiome-based immunotherapeutics that affect or mitigate GVHD and enumerate the emerging strategies for the regulation of dysbiosis and microbial metabolites in the regulation of GVHD.

### Gut Microbiota and Intestinal Epithelial Cells

**Figure 1:**
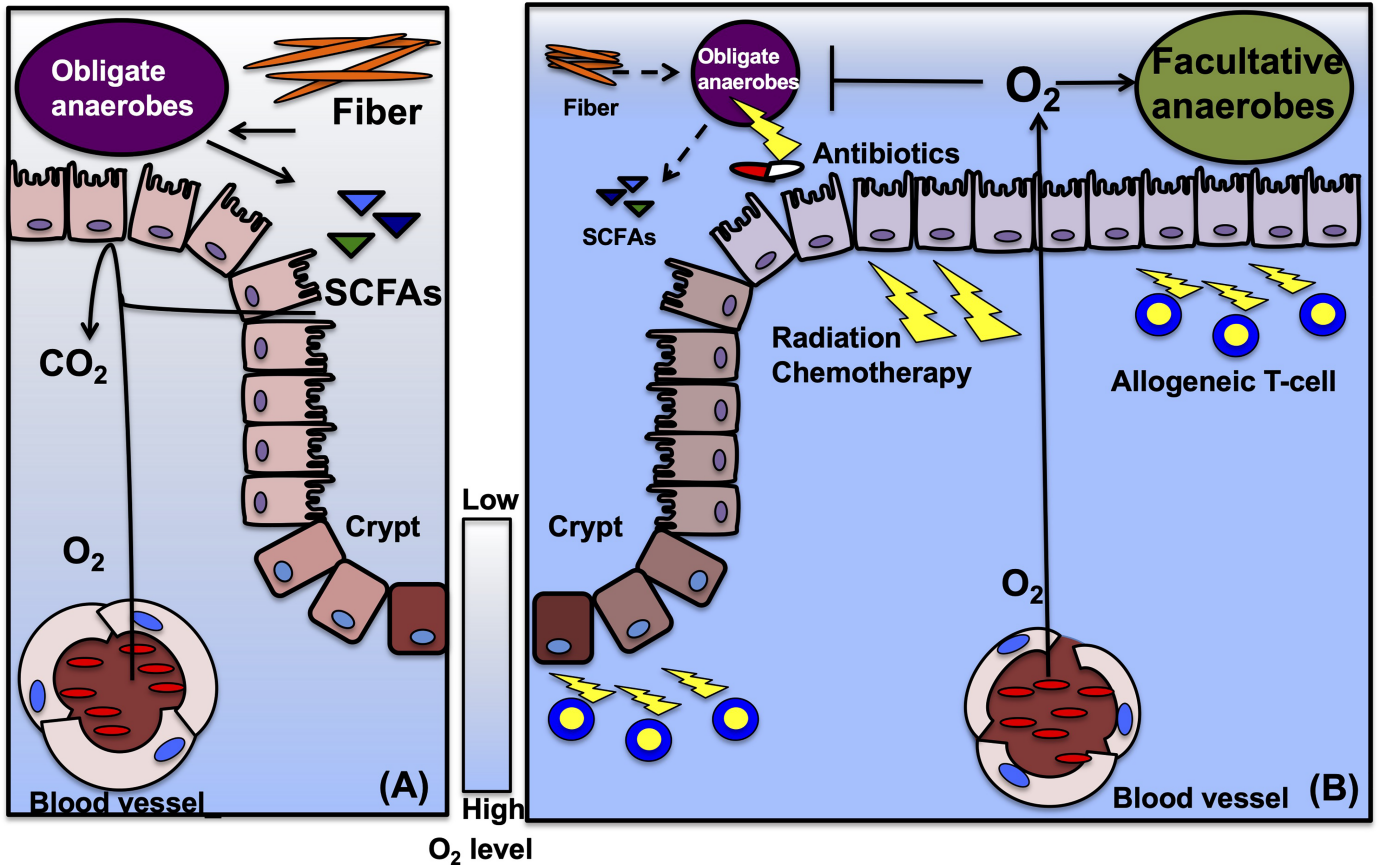
Suggestive mechanisms of epithelial metabolism and gut microbiota in HSCT. **(A)** Before HSCT, gut microbiota, especially obligate anaerobic bacteria, ferment fiber from diet into metabolites, such as SCFAs. Intestinal epithelial cells (IECs) metabolize short chain fatty acids (SCFAs) and consume oxygen to produce energy. High oxygen consumption limits the oxygen diffusion into the lumen and maintains hypoxia in the lumen. Hypoxic condition enables obligate anaerobes to keep growth. The scale bar indicates oxygen (O_2_) levels, usually between 3% to 10% in normal intestines. **(B)** During HSCT, conditioning treatments including irradiation and high-dose chemotherapy damages IECs, and injured IEC decreases oxygen consumption. Decreased dietary fiber and antibiotics treatments during HSCT disrupt microbiota composition, especially reduce obligate anaerobes, and deplete the fermentative products. Reduced SCFAs limit oxygen consumption to produce energy in IECs and permit oxygen diffusion into the gut lumen. Elevated oxygen concentration drives an expansion of facultative anaerobic bacteria and might lead to a contraction of obligate anaerobes. In GVHD, allogeneic T-cells promote IEC injuries and require antibiotics treatments, that could enhance and maintain dysbiosis in the gut.

A close relationship between microbiota and human health has been investigated. Microbiota refers to the community comprising trillions of microorganisms, including bacteria, fungi, and viruses, that symbiotically colonize the human body, most members of which reside in the gut and are largely nonpathogenic anaerobic commensal bacteria (8). This balance is finely tuned in the gastrointestinal (GI) tract and influenced by the environment, diet, and host factors, including host physiology (9). The mammalian GI tract is a relatively hypoxic tissue and has a steep oxygen (O_2_) gradient between the O_2_-rich lamina propria and the gut lumen, which is dominated by anaerobic organisms (10). Aerobic and facultative anaerobic bacteria have been suggested to consume oxygen in the distal intestine and maintain hypoxia in the lumen, leading to the colonization by strict anaerobes and the production of short-chain fatty acids (SCFAs) (11). Complex dietary carbohydrates (fiber) are broken down by these bacteria into digested fermentation products, that are absorbed by the host and utilized for host nutrition, immune development, and niche protection against enteric pathogens (12–19). Members of *Clostridia* and *Bacteroidia* are the obligate anaerobic bacteria dominating in the colon and capably digest any carbohydrate complex into fermentation products (20, 21). Facultative anaerobic bacteria such as *Proteobacteria* are not specialized in consuming fiber, and rather interfere with host nutrition by changing metabolites to carbon dioxide under oxygen (22, 23). Fermented metabolite effects have been observed in a neonatal mouse model with *Clostridia* species colonization that protected neonatal mice form virulent pathogens (24). Intestinal epithelial cells (IECs) are continuously renewed from the crypts where intestinal stem cells (ISCs) are located. ISCs divide and differentiate into transit-amplifying (TA) cells, IECs, enteroendocrine cells and goblet cells (25). In contrast to TA and ISCs that utilize glucose to obtain energy through glycolysis, IECs produce energy *via* mitochondrial β-oxidation of fatty acids and oxidative phosphorylation, both of which require oxygen (26, 27). Butyrate is a favored metabolic substrate that maintains epithelial energy homeostasis (26). Despite low O_2_ conditions, high butyrate levels result in the downregulation of the glycolytic pathway and the upregulation of mitochondrial respiration (28). This drastic change of energy production was triggered with the expression of peroxisome proliferator-activated receptor-γ (PPAR-γ), which is a nuclear receptor synthesized in differentiated IECs of rodents and humans (29). The energy metabolism in IECs consumes a high amount of oxygen, leading to low oxygen pressure in the lumen (30). This oxygen consumption in IECs permits epithelial hypoxia and helps to maintain anaerobic bacterial flora in the intestinal lumen (31). The hypoxic environment enables obligate anaerobic bacteria that produce carbohydrate metabolites to colonize and provide benefits to the host. Epithelial metabolism of SCFA is a primary determinant of ‘‘physiologic hypoxia’’ in the mucosa with O_2_ consumption and hypoxia-inducible factor (HIF) stabilization that promotes barrier function (32). Therefore, IECs are uniquely adapted to this hypoxic environment, and cells programmed by “physiological hypoxia” have been shown to tonally regulate barrier function (33). These mechanisms indicated that IECs influence the shaping of beneficial microbiota, and those microbiotas bring metabolic profits to the host. In this process IECs play a central role in maintaining gut homeostasis (**Figure 1**).

The IECs and gut microbiota function in two distinct manners: protection against ingested pathogens and induction of tolerance to beneficial commensals. The intestinal immune system matured by microbiota stimulates the mucosa-producing cells (i.e., Paneth cells and goblet cells) to secrete antimicrobial peptides, antibodies, and mucin to form intestinal physical barriers, such as the mucus layer and tight junctions that regulates the relationship between the microbiota and the host (34). Germ-free mice have distinct gut features, such as altered intestinal morphology and slower turnover of IECs than conventional mice (35). After exposure to exogenous commensal bacteria, germ-free mice responded to intestinal bacteria. Four days post-exposure, innate immune responses and structural changes in the intestinal epithelial crypts were observed, i.e., increased TNF-α and interferon-γ (IFN-γ) expression and upregulation of MHC class I molecules (35). IECs in SPF mice also expressed MHC class II molecules that were deficient in germ free mice (36). Under the inflammatory status such as GVHD, gut microbiome stimulated IL-12 production from myeloid cells, resulting in increased expression of MHC class II on IECs through IFN-γ produced from lamina propria lymphocytes. In summary, the intestinal environment shapes and maintains the microbiota. Also, microbiota and those metabolites play important roles on both the function and metabolism in IECs.

### Mechanism of Dysbiosis in Hematopoietic Cell Transplantation

Associations between the microbiota and HSCT outcomes have been extensively studied in the 1970s (37). According to these early studies, specific pathogen-free mice treated with antibiotics or germ-free mice that received HSCT developed less severe GVHD than their respective controls. These attempts were validated in HSCT patients with gut decontamination by antibiotics (38). Recently, broad-spectrum antibiotics that target anaerobic pathobionts have been revealed to increase GVHD-related mortality in mouse and human (39). Therefore, some antibiotics that perturb microbial composition were unsuitable for patients in HSCT settings. The microbial compositions of various anaerobic commensal intestinal bacterial species were robustly disturbed and reduced due to chemo/radiotherapy, antibiotics, or HSCT itself in patients (40). Decreased diversity and expansion of specific bacteria, including *Enterococcus* spp., were reported as risk factors that resulted in poor outcomes after HSCT (41). The mono-domination of *Enterococcus* was significantly associated with severe GVHD and treatment related mortality (TRM) in mice and human studies (42). These studies indicate the causative role of *Enterococcus* in the pathogenesis of GVHD. However, the mechanism of dysbiosis post-HSCT remains unclear (7, 43). Before and after HSCT, patients receive various interventions that are likely to contribute to the microbial dysbiosis. Importantly, in a mouse model of acute GVHD exposure to a GVHD-associated gut microbiome pre- and/or post- HSCT accelerated GVHD development and severity demonstrating the complex relationship between GVHD and microbiota (44). These factors could affect the intestinal microbial composition (**Figure 1**).

### Radiotherapy

As a part of allo-HSCT, systemic irradiation is frequently used to kill fast-dividing leukemia/lymphoma cells and host myeloid cells/lymphocytes. However, radiation causes intestinal damage, and the potential correlation between dysbiosis and radiation-induced damage has been revealed (45, 46). Radiation induces oxidative stress *via* the generation of reactive oxygen species (ROS), including hydroxyl radical (OH), superoxide anion (O^2−^), and hydrogen peroxide (H_2_O_2_), resulting in the activation of cyclooxygenases (COX), nitric oxide synthases, lipoxygenases, and nicotinamide adenine dinucleotide phosphate oxidases. These products of oxidative stress cause DNA damage, inflammation, and apoptosis in IECs and affect the gut microbiota composition through homeostatic disturbances (45). The relationship between radiation enteritis and dysbiosis was observed in patients receiving radiotherapy. Relatively high abundances of *Proteobacteria* and *Gammaproteobacteria*, characterized by increased oxidative stress resistance in *Enterobacteriaceae, Phyllobacteriaceae*, and *Beijerinckiaceae*, and low abundance of *Bacteroides* with decreased *Bacteroidaceae* and *Ruminococcaceae* that were sensitive to oxidative stress, were detected (46). Recently, a multi-omics study reported *Lachnospiraceae* and *Enterococcaceae* as radioprotective microbes and elevated SCFA levels due to irradiation damage in mice receiving irradiation (47). *Bifidobacterium* can utilize indigestive fibers, such as fructose oligosaccharides (FOS), inulin-type fructans (ITF), and xylo-oligosaccharides (XOS), and cross-feed with lactate-converting bacteria and butyrate-producing bacteria, i.e., *Eubacterium hallii* and *Anaerostipes caccae* (48). Although *Bifidobacterium* cannot produce butyrate, it is associated with increased abundance of butyrogenic bacteria in a cross-feeding manner (49). Moreover, *Eubacterium hallii* L2-7 and *Anaerostipes caccae* L1-92 could not grow in starch but grew upon co-culture with *Bifidobacterium adolescentis* L2-32 to produce butyrate (50). Collectively, the abundance of specific bacterial species is reduced and eventually lost post irradiation before allo-HSCT.

### Chemotherapy

Elaborating the purpose of repeated intensive chemotherapy before allo-HSCT reduces microbiota diversity in both human and mice and leads to the expansion of *Escherichia Coli* and *Enterococcus* spp (51). During allo-HSCT, disrupted gut microbial composition and diversity, including decreased abundances in *Bifidobacterium*, butyrate-producing *Faecalibacterium* and *Lachnospiraceae*, were highly associated with transplant-related mortality (7). Methotrexate treatments caused a significant reduction in the number of *Bifidobacterium* and *Lactobacillus* species and *Escherichia coli* in children with leukemia (52). Etoposide exhibits broad bacterial inhibitory activity that changes microbial composition *in vitro* (53). In leukemia patient data, it is suggested that chemotherapy induces dysbiosis (51). Healthy volunteers in the Human Microbiome Project (HMP) and patients with acute myeloid leukemia in the pre-induction phase showed no significant differences in terms of microbial diversity. After neutrophil recovery, microbial diversity in the leukemia group was significantly decreased and this dysbiosis was associated with an increased risk of infections and the use of broad-spectrum antibiotics. Within 3 months of anti-anaerobic antibiotic administrations including carbapenem and piperacillin-tazobactam, a significant reduction in microbial diversity was also observed before allo-HSCT in the recipients (54). Multiple intensive chemotherapies for acute leukemia patients also disrupt the microbial composition and promote the outgrowth of pathogenic bacteria such as *Enterococcus* (55). Another study demonstrated that pre-transplant microbial composition differed from the healthy controls due to decreased abundances of beneficial bacteria, *Bifidobacterium*, *Faecalibacterium*, and *Lachnospiraceae* (56). Overall, these findings suggest that pre-transplant microbe disruption increased HSCT comorbidity. However, the mechanisms of dysbiosis onset and the disruption of the microbiota by chemotherapy are still unknown. Furthermore, these studies could not separate the effects of HSCT from those of antibiotics (39).

### Antibiotics

Chemotherapies for hematological malignancies cause high-grade neutropenia and require systemic antibiotic administration to prevent or treat life-threatening bacterial infections. The influence of antibiotics on the microbial composition should be accounted for. Recent microbiome studies have observed undesirable consequences in dysbiosis, including antibiotic resistance, pathogenic bacteria dominance, transient or profound loss of microbial diversity, increased susceptibility to infection, and the risk of recurrent infections, after antibiotic usage in cancer patients (57). However, immunocompromised individuals are at high risk of intestinal infections, which are difficult to treat without using broad-spectrum antibiotics (58). As reported by various studies, oxygen-tolerant species, *Enterococcus, Enterobacteriaceae, Klebsiella* spp., and *Viridans streptococci*, are most commonly translocated from damaged intestinal mucosal barrier due to chemotherapy, irradiation, and antibiotics to the bloodstream (59). Prophylactic antibiotics, such as quinolones, are usually utilized to prevent infections in patients receiving HSCT. Once they develop fever or infections, broad-spectrum antibiotics, such as piperacillin-tazobactam or meropenem, are prescribed (60). Compared to pre-transplant conditions, decreased *Firmicutes* and *Actinobacteria* and increased *Bacteroides* and *Proteobacteria* have been detected at 1week post-conditioning (61). Notably, the use of rifaximin could preserve gut beneficial commensals and improve patient outcomes (62). HSCT patients receiving rifaximin showed significantly lower TRM, prolonged OS, and a tendency for low acute GI-GVHD rates than historical controls. These observations remained unknown even in patients who developed neutropenic fever and received systemic antibiotics without an increase in sepsis rates or pathogenic bacterial colonization. Efforts to investigate and identify the antibiotics that could spare microbial compositions to reduce acute GI-GVHD and adverse outcomes have been attempted (63). A study in patients from Canada also assessed the effects of prophylactic and therapeutic antibiotic administration before day 0 of HSCT (64). The antibiotic-receiving group had a significantly higher incidence of acute GVHD (aGVHD) and shorter survival. The early administration of systemic antibiotics before engraftment was also associated with low 3-IS levels and reduced Clostridia abundance in the intestines, leading to higher TRM rates than those without systemic antibiotics before engraftment (65). These studies suggest the association of antibiotic usage affecting microbial diversity and GVHD-related outcomes. Piperacillin-tazobactam and meropenem, known as broad-spectrum antibiotics that are also effective against anaerobic bacteria, caused decreased microbiome diversity during transplantation in mice and humans (39, 66). In mouse models, these antibiotics lead to microbial injury with loss of colonic mucosa and intestinal barrier function caused by mucin-degrading bacteria, *Akkermansia muciniphila* (39). The load of anti-anaerobic antibiotic usage was correlated with a significant decrease in anti-inflammatory Clostridia (AIC) abundance and aGVHD in pediatric HSCT patients (67). This decrease was recovered by the administration of AIC after clindamycin treatment and improved survival in a mouse GVHD model (67, 68). Among the broad-spectrum antibiotics administered during HSCT, meropenem showed significantly decreased microbial diversity and a higher rate of GI-HVHD onset compared to no antibiotics, but cefepime did not change the diversity with a trend of increased GI-GVHD onset rates (69). In a pediatric study, anti-anaerobic antibiotics resulted in a significant decrease of SCFA-producing bacteria, especially butyrate-producing commensals and butyrate levels in the luminal content. These patients developed aGVHD with low butyrate levels in stools compared to patients without GVHD (70). Single bacterial taxa dominated by Enterococcus and Streptococcus was observed in two-thirds of patients with bloodstream infections around the time of engraftment (71). The significant elevation of enterococcus expansion was increased by metronidazole administration, and VRE bacteremia was significantly increased (3-fold and 9-fold, respectively). Integrating these studies, although antibiotic prophylaxis and systemic administration clearly improved TRM, especially infection-related mortality, we need comprehensive choices of antibiotic usage around HSCT periods comparing weights of disease status, donor sources, history of antibiotic usage, status of microbial injuries, and other risk factors.

## Metabolic Changes in Colonocytes

IECs might control the homeostasis that shapes the microbiota to be beneficial (72). Dysbiosis that caused increased abundance of facultative anaerobic bacteria, is observed in the population consuming a high-fat Western-style diet, patients with inflammatory bowel disease, colorectal cancer, irritable bowel syndrome or necrotizing enterocolitis (73–80). It has been proposed that shift of microbial community from obligate to facultative anaerobic bacteria that can utilize oxygen could be associated with a disruption in aerobiosis, a concept “oxygen hypothesis” in mouse inflammatory bowel disease models (81, 82). This microbial shift might be associated with the colonocyte metabolic dysfunction (31). Disruption of gut homeostasis was observed in antibiotic treatment that altered IECs metabolism by depleting microbes that produce SCFAs in a mouse colitis model (19, 32). Decreased SCFAs increases the inflammatory tone of the colonic mucosa in mice (83). In the animal model, elevation of inflammatory signals shifts the metabolism in IECs toward an aerobic glycolysis, reduced oxygen consumption and high glucose consumption and high lactate release (84). These metabolic changes result in a loss of epithelial hypoxia (32). Increased oxygen concentration elevates the amount of oxygen in mucosal surface, therefore drives an expansion of facultative anaerobic bacteria (85). These associations between metabolic condition in IECs and dysbiosis are not validated in GVHD settings and the future investigations are required.

### Butyrate and Lactase Pathway

Recent evidence has suggested that dysbiosis post allo-HSCT are related to the butyrate and lactase pathways (86). Butyrate is produced from non-digestible fiber by anaerobic bacterial species, including those belonging to *Firmicutes*, *Lachnospiraceae*, *Ruminococcaceae*, *Lactobacillus* spp., and *Bifidobacterium adolescentis*. Other bacterial phyla including *Bacteroides*, *Actinobacteria, Fusobacteria*, and *Proteobacteria* are also potent butyrate producers (87). These bacteria are sensitive to oxidative stress caused by chemotherapy and irradiation administered before HSCT, leading to their reduced abundances and the subsequent decrease in butyrate volume in the lumen. At the intestinal level, butyrate is a protective molecule against inflammation, a histone deacetylase inhibitor, and an energy source for epithelial cells (68, 88). Therefore, decreased butyrate concentration led to GVHD augmentation due to the disturbance of intestinal homeostasis and the activation of inflammatory environment in the intestine. Another mechanism that causes dysbiosis post-HSCT in GVHD patients involves the domination of the lactic acid bacteria (LAB) *Enterococcus faecium* in the gut (42). In GVHD patients, metabolism of lactose and galactose is impaired by the overexpression of enzymes and lactases (89). In adults, lactase is mainly produced by the microbiota, including species belonging to *Actinobacteria, Proteobacteria*, and *Firmicutes* (90). Each LAB has distinct enzymatic characteristics and behaves differently depending on each feature. LABs are mainly gram-positive, acid-tolerant bacteria from the genera *Lactobacillus, Enterococcus, Streptococcus, Pediococcus, Lactococcus*, and *Oenococcus* (91). They produce lactase that catalyzes the conversion of carbohydrates (such as lactose, glucose, sucrose, and galactose) into lactic acid and facilitates lactose absorption (92, 93). The reduction in the number of commensal LAB mediated by conditioning chemotherapy, radiotherapy, and antibiotics post-HSCT leads to high lactose concentrations. For example, *Enterococci* are a part of the normal intestinal microbiota and can cause clinical problems. Particularly, *Enterococcus faecium* and some *Enterococcus faecalis* encode genes related to lactose and galactose metabolism (42). *Enterococcus faecium* metabolizes citrate and lactose to produce lactate, whereas *E. faecalis* can produce superoxide and H_2_O_2_ that damage colonic epithelial cells (94, 95). These enterococci require lactose for growth *in vitro* (lactose auxotroph) and are associated with intestinal inflammation and damages (96). These pathways were observed following the expansion of *Enterococci* early after HSCT in patients with the increased risk of HSCT-related mortality (43). Butyrate and lactase pathway could be a target for treating dysbiosis in HSCT. The analysis of these pathways for TRM post HSCT has been reviewed in detail previously (89).

## Microbial Metabolites in GVHD

The intestinal microbiota produces various microbial metabolites that maintain intestinal homeostasis (97, 98). These metabolites affect both IECs and immune cells in the intestine to maintain intestinal barrier function and host immune responses (97). Changes in microbial composition led to poor HSCT outcomes; however, to date, there is no conclusive evidence that changes in gut microbial metabolites affect GVHD severity and TRM.

### Short-Chain Fatty Acids

**Figure 2:**
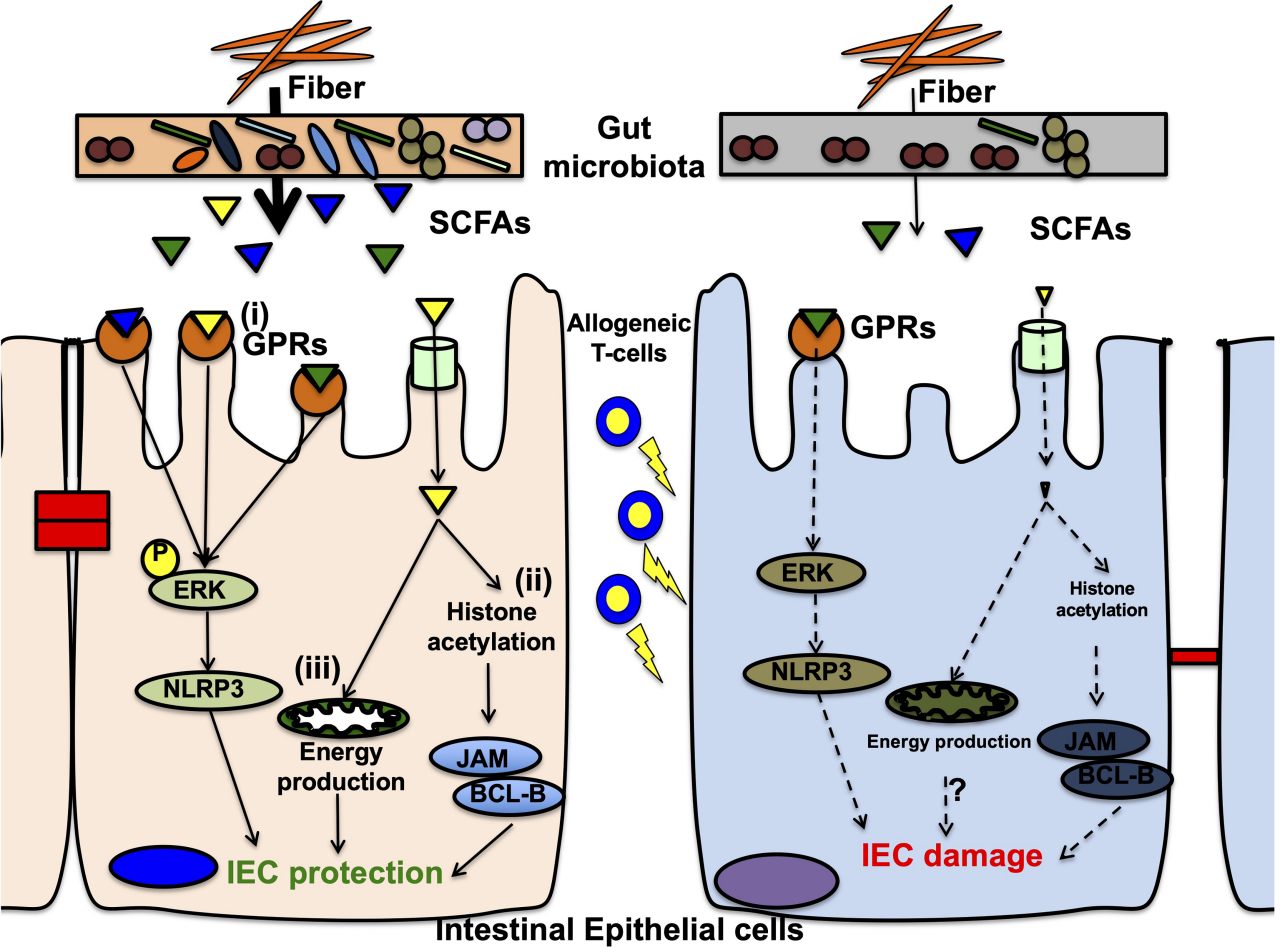
Mechanisms of short chain fatty acids on intestinal epithelial cells in the pathophysiology of gastrointestinal GVHD. In the normal condition, short chain fatty acids (SCFAs) that are metabolites from microbial fermentation of dietary fibers, protect intestinal epithelial cells (IECs) in at least 3ways. (i) G-protein-coupled receptors (GPRs) on IECs, especially GPR43, sensor SCFAs and signal ERK phosphorylation leading to subsequent NLRP3 inflammasome activation, that promotes IEC integrity. In graft-*versus*-host disease (GVHD), decreases of GPRs on IECs and reduced SCFAs from dysbiosis diminish intracellular signaling *via* ERK-NLRP3 pathway, resulting in IEC damages. (ii) One of SCFAs, butyrate acts as a histone deacetylase inhibitor, and increases various target gene expressions, including anti-apoptotic BCL-B and the junctional protein JAM (junctional adhesion molecule). These gene expressions result in decreased IEC apoptosis and enhanced junctional integrity, leading to IEC protection. In GVHD, reduced butyrate fails to protect histones from deacetylation and decreased target gene expression, resulting in low resistance against allogeneic T-cell injuries. (iii) Butyrate activates peroxisome proliferator-activated receptor-γ (PPAR-γ) signaling in IECs and promotes mitochondrial ß-oxidation that produce enough amount of energy to protect and maintain IECs from cellular damages. In GVHD, reduced butyrate might not activate PPAR-γ, resulting in mitochondrial dysfunction for energy production that are inappropriate for IEC protection against allogeneic T-cell injuries. This mechanism is studied in inflammatory bowel disease but not validated in GVHD. Further investigations are required.

SCFAs, including acetate, butyrate, and propionate, are products of fermented carbohydrates by anaerobic commensal bacteria *Clostridia* spp (99). SCFAs have multipotent effects on both IECs and intestinal immune cells (**Figure 2**). Particularly, butyrate serves as an energy source for IECs as demonstrated by reduced autophagy in SPF mice compared to germ-free mice (26). In addition, SCFAs preserve the intestinal mucosal barrier by supporting goblet cells *via* upregulating mucin-related genes. The supplementation of SCFA-producing bacteria in germ-free rats showed goblet cell maturation (100–102). SCFAs also maintain the IEC barrier integrity that prevents pathogenic bacterial translocation (103). Furthermore, they play an important role in innate immunity, neutrophils, mononuclear cells, macrophages, and dendritic cells and act as histone deacetylase (HDAC) inhibitors with anti-inflammatory effects (99, 101, 102). SCFAs also affect Tregs to promote differentiation and anti-inflammatory responses in a mouse colitis model (17). Moreover, anti-inflammatory IL-10 production from Foxp3-positive Tregs *via* HDAC inhibition was observed in germ-free mice with SCFA supplementation (18, 104). A high-fiber diet and acetate activated the inflammasome and promoted IL-18 production, which modulated epithelial repair in murine colitis model (105). Butyrate also increased IL-18 expression in IECs and IL-10 expression in DCs and macrophages, thereby inducing Treg differentiation in mice (106). Recently, a metabolomic analysis of murine intestine and contents revealed a significant reduction in butyrate in the IECs from GVHD mice and that the supplementation of butyrate or butyrate-producing bacteria ameliorated acute GVHD *via* HDAC inhibition and gut integrity maintenance (68, 107). Butyrate and propionate directly protected IECs *via* inflammasome activation through SCFA-sensing receptors in murine GVHD model (108). In humans, small clinical studies have found that the high fecal butyrate and propionate concentrations are decreased in acute GVHD patients and that high circulating butyrate and propionate concentrations in the blood are associated with protection from chronic GVHD (70, 107). Other studies examined fecal SCFA concentrations after allo-HSCT and reported that the concentrations of butyrate and other SCFAs correlate with the abundance of butyrate-producing bacteria in the intestinal microbiota and are higher in patients with resistance to lower tract respiratory infections (109). However, the effect of SCFAs on GVHD needs to be examined in patients after allogeneic HSCT.

During HSCT, recipients have problems that limit oral intake due to mucositis and receive total parenteral nutrition (TPN). In both mice and humans, TPN skewed immune responses to pro-inflammatory conditions (110, 111). Positive nutritional support using sucrose improved hematopoietic recovery compared to antibiotic-treated recipients in mice (112). These studies overall suggest the potential of specific diet elements (prebiotics) during HSCT. Resistant carbohydrate supplementation can stimulate SCFA production in the intestine as the microbiota can metabolize resistant starch (RS) (113). Among various formulations of RS, that derived from potatoes was identified as a potent candidate for increasing fecal butyrate levels in healthy adults (114). Remarkably, RS administration to allo-recipients around HSCT periods was demonstrated as feasible and safe and lead to increased fecal butyrate levels concomitant with high RS degrading and butyrate-producing bacteria in a human pilot study (115). However, the clinical data on the effects of prebiotics in acute GVHD are limited. Inulin and fructo-oligosaccharides have potential as prebiotics in inflammatory bowel disease patients as they led to the expansion of intestinal microbial diversity (116, 117). To determine the exact role of nutritional modulation using RS in the microbiome and metabolites affecting GVHD severity, clinical trials are currently ongoing (www.clinicaltrials.gov: NCT02763033). A clinical trial that evaluating the safety and tolerability of fructo-oligosaccharides in HSCT patients (NCT02805075) is also underway.

### Bile Acids

Bile acid is a microbial metabolite affected by intestinal microbial composition (118). Primary bile acids are generated in the liver *via* cholesterol catabolism. These primary bile acids are conjugated to glycine and taurine in the final step of synthesis and are secreted into the intestine. Most bile acids are taken up by the IECs and transported to the liver (119). Secondary bile acids are converted by microbial enzymes from primary bile acids that were not absorbed. Two main receptors, the nuclear farnesoid X receptor (FXR) and G protein-coupled bile acid receptor 1 (GPBAR1; TGR5), are known. FXR-deficient mice showed abnormal IEC functions, thus causing bacterial translocation from the intestine (120). TGR5 signaling from bile acids activates macrophages and inhibits the production of pro-inflammatory cytokines TNF-α and IL-1 by inhibiting the nuclear factor-κB (121). Dietary fat-induced taurocholic acid in IL-10-deficient mice enhanced colitis in mice by altering the intestinal microbiota and pro-inflammatory Th1 responses (75). While bile acids are not well absorbed in patients with intestinal GVHD and serum sample analysis of allo-HSCT patients revealed altered bile acid concentrations, ursodeoxycholic acid, a secondary bile acid, is clinically used to reduce hepatic complications and GVHD (122–124). In a recent study, tauroursodeoxycholic acid, another bile acid, reduced the experimental murine GVHD severity (124). Tauroursodeoxycholic acid decreased antigen presentation on IECs, and the apoptosis of IECs without affecting the microbial composition and graft-*versus*-leukemia/lymphoma (GVL) effects. Taurine is a metabolite related to bile acids that stimulates the Nod-like receptor family protein domain containing 6 (NLRP6). NLRP6 stimulation leads to innate immune cell activation, increased apoptosis/necroptosis, inflammasome-related cytokine production, and promotion of intestinal epithelial integrity (125). Taurine has been reported to stimulate NLRP6 in IECs and augment GVHD severity in mice (126). These data suggest that bile acids are promising microbial metabolites that can be used to reduce GVHD severity.

### Aryl Hydrocarbon Receptor Ligands

Aryl hydrocarbon receptor (AhR) modulates mucosal immune responses *via* the ligand-activated transcription factor. AhR ligands are produced from the endogenous and exogenous pathway in the body. In an endogenous pathway, they are produced through intestinal microbial metabolism. *Lactobacilli*, *Fusobacterium*, *Bacteroides* and *Enterococcus faecalis* convert the amino acid, tryptophan, into AhR ligands, such as indole-3-aldehyde and its derivatives (127, 128). In a human GVHD cohort, preserved microbial diversity and *Clostridia spp* were correlated with 3-IS concentrations in the urine (129). In serum metabolic comparisons of HLA identical donors and recipients, indolepropionate that was derived from microbiota, was a smaller amount in recipients at the time of GVHD onset (123). 3-IS is also known as a uremic toxin associated with adverse outcomes in renal disease patients. In this setting, monocytes respond to 3-IS through AhR pathway and release TNF-α, resulting in the pro-inflammatory condition with endothelial damage leading to cardiovascular disease (130). AhR ligands maintain IEC barrier functions. AhR-deficient mice have decreased levels of anti-microbial peptides and intestinal epithelial lymphocytes and reduced IEC turnover with altered microbial composition in mice (131). AhR also regulates innate immunity in the intestine by expanding the IL-22-producing retinoic acid receptor-related orphan receptor γt (RORγt)+ group 3 innate lymphoid cells (ILC3s) (132), thereby affecting adaptive immunity (133). In murine GVHD models, a few investigations were reported. Recipient mice that exposed to lethal irradiation or chemotherapeutic conditioning regimens had lower urinary concentrations of 3-IS (134). The supplementation of indole-3 carboxaldehyde, an indole derivative, ameliorated gut epithelial damages with reduced inflammatory cytokines, leading to improved survival. In murine GVHD models, AhR-deficient T-cells ameliorated GVHD through the expansion of peripherally induced Tregs (135). AhR ligand levels were decreased in patients at the onset of GVHD (123). However, the mechanism by which the AhR pathway affects GVHD in mice and humans remains unknown.

### Tyrosine Derived Metabolites

Tyrosine derived metabolites are produced from tyrosine fermentation by microbiota in the large intestine (136). Tyrosine is one of the non-essential amino acids that is involved in catecholamine synthesis (137). The function of tyrosine has been well studied in brain physiological and pathological conditions (138). In a mouse GVHD model, the metabolic profiles in recipient mice have been determined (139). The low concentration of tyrosine in the gut was observed in GVHD mice, and the tyrosine derived metabolites were decreased in the mice that has less *Blautia* and *Enterococcus*. Dietary supplementation of tyrosine ameliorated GVHD and restored the microbial diversity. In GVHD patients, the metabolite markers in the serum have been investigated (140). Alteration of tyrosine derived metabolites, such as p-cresol sulfate and 3-phenylpropionate, were identified. Also, lysine and phenylalanine were suggested as the altered metabolites. To elucidate the importance of these metabolites, further research is necessary.

### Choline Derived Metabolites

Choline derived trimethylamine N-oxide (TMAO) results from oxidation by hepatic flavin monooxygenases of trimethylamine (TMA) that is a microbial metabolite of choline and other choline containing compounds in the diet (141). TMAO is well known to play a role in the initiation of atherosclerosis and thrombosis in vascular inflammation and endothelial function (142). TMAO and choline induced the GVHD progression in a murine GVHD model (143). TMAO and choline induces M1 macrophages and M1-like cytokines in tissues and bone marrow *via* NLRP3. TMAO are produced by the wide range of bacteria, *Anaerococcus hydrogenalis, Clostridium asparagiforme, Clostridium hathewayi, Clostridium sporogenes* (144).

### Riboflavin (Vitamin B2) Derived Metabolites

Riboflavin derived metabolites are also known to have an important role in the GVHD pathogenesis. Various bacteria including *E. coli, Staphylococcus aureus and Pseudomonas aeruginosa*, produce these metabolites (145). These metabolites expand the numbers of mucosal associated invariant T-cells (MAIT) (146). Vitamin B2/B9 derived metabolites are presented by MR1, the MHC class I-like molecule, to MAIT cells producing IFN-γ, IL-17 and antibacterial products. In mouse and human GVHD, studies involving riboflavin derived metabolites and GVHD have been accumulating, but there is still no direct evidence that the riboflavin concentration in feces has a direct impact on HSCT complications and outcomes (147–150). In cord blood transplantation, microbial expression of enzymes in the riboflavin synthesis pathway was associated with greater MAIT reconstitution after HSCT (151).

### Polyamines

Polyamines are polycationic molecules found in the gastrointestinal tract and are produced by microbial metabolism. They have various biological functions, including IEC barrier function, innate immunity, pro-inflammatory cytokines, and adaptive immunity (152). Spermine, a polyamine, preserves TGF-β and IL-10 production by inhibiting pro-inflammatory cytokine production in activated macrophages (153). Moreover, the amino acid arginine is metabolized to produce polyamines and decreases the production of pro-inflammatory cytokines when administered with *bifidobacterial LKM512* (154). The oral supplementation of spermine or spermidine promoted intraepithelial CD8^+^ T-cell maturation and CD4^+^ T-cell increase in the lamina propria and B cell increase in the rat spleen (155). However, the effects of polyamines on GVHD have not yet been well studied. The oral microbiome is suggested to play a role in pathological conditions and mucositis in allo-HSCT settings (156, 157). During allo-HSCT, oral microbiota derived metabolites are altered in patients with severe oral mucositis, and reduction of N-acetylputrescine and agmatine, metabolites involved in the polyamine pathway, were reported from salivary metabolic analysis in patients (158).

### Fecal Microbiota Transplant

Alterations in intestinal microbiota composition and its contribution to allo-HSCT outcomes have been studied to determine the role of the intestinal microbiota in acute GVHD severity. The strategy of manipulating or improving dysbiosis post allo-HSCT *via* fecal microbiota transplant (FMT) were introduced in the HSCT field because FMT treatment for *Clostridium difficile* infection in non-HSCT settings has been successfully reported (159). Recently, the utility of FMT in patients with refractory GVHD after allo-HSCT has also been reported (160). After FMT treatment, dysbiosis improvement was observed as evidenced by increased beneficial bacteria and resolution of clinical symptoms (161). The effects of FMT from third parties were also assessed in a pilot study, and the feasibility and effects on microbiome diversity in recipients have been reported (162). An increase of *Clostridiales* abundance was correlated with a significant increase of 3-IS concentration in the urine in this study. In a randomized trial involving autologous FMT early after allo-HSCT without broad-spectrum antibiotics, the expansion and reestablishment of gut microbiota diversity in autologous FMT recipients was reported (163). Although only a limited number of studies regarding FMT and GVHD treatment have been conducted, they have reported similar results (164). FMT products were freshly processed or frozen until subsequent use. The routes of administration mainly include oral in packed capsules, nasogastric/nasoduodenal tubes, or enema. Most FMT recipients were treated using third-line or more therapies, whereas some received second-line therapy after steroid failure. Complete response rate was high upon treatment with second-line therapy. Responses to treatment were observed within an average 14 days, with a median of two FMT administrations. Changes in the stool microbiome were analyzed using bacterial sequences. FMT recipients had increased diversity and enrichment of *Bacteroides, Lactobacillus, Bidifobacterium*, and *Faecalibacterium* compared with pre-FMTs (160). These changes were observed only when anti-anaerobic systemic antibiotics were discontinued; however, the fourth generation cephem did not affect the efficacy of FMT.

The gut microbiome comprises communities of bacteria, fungi, and viruses that affect each other. Similar to bacterial dysbiosis, alterations in the gut viral community are also associated with gut GVHD (165). For instance, human herpes virus 6 was detected in the serum, and picornavirus in the stool of acute GVHD patients (166). The intestinal fungal composition (mycobiome) after HSCT remains not well investigated. The expansion of pathogenic *Candida* species was associated with a substantial loss of bacterial diversity, especially that of anaerobes, and increased the risk of fungal bloodstream infections (167). There is a paucity of data regarding the changes in the mycobiome and virome in allo-HSCT post-FMT (168). In a GVHD patient with repeated FMT treatments, longitudinal studies observed dynamic changes in the microbiome, mycobiome, and virome in the stool. In contrast to the expansion of intestinal microbial composition post-FMT, the gut mycobiome was first expanded and decreased after multiple FMTs. The gut virome community varied substantially over time with a stable increase in diversity. Furthermore, increased microbial diversity post-FMTs was consistently reported; however, the suggested mechanisms need to be elucidated, including the changes in the microbial metabolites and the key bacterial strains.

Overall, most studies have concluded that FMTs are generally safe and effective for steroid-refractory GVHD patients. However, infectious complications and deaths have been reported, including death due to transmitted drug-resistant bacteria from the FMT donor, bacteremia not from FMT products, diarrhea due to norovirus in the FMTs, and other infections, and were attributed to the immunocompromised states of the patients. Importantly, critical complications related to the recipient death have been reported, such as the transmission of extended spectrum beta-lactamase producing *Escherichia coli* that was proven using genomic sequencing from FMT products (169). Before the clinical application of FMTs, the preparation of FMT products should be standardized, and universal stool banks are warranted (170). The precise practical aspects of FMT treatments have been reviewed in detail elsewhere (171).

## Future Perspectives

The effects of dysbiosis post-HSCT on GVHD have been investigated. Currently new studies on mycobiome and virome in the intestine post-HSCT are focused because intestinal environments were composed of various microorganisms other than microbiota. As viruses and fungi are also part of the gut microbiome, they are expected to provide a deeper understanding of the connection between the microbiome and GVHD. Microbial-derived metabolites, such as SCFAs and indoles, play critical roles in promoting intestinal homeostasis to overcome unwanted antibiotic influences and unfavorable outcomes after HSCT. There are various targets that can affect the results of HSCT, including microbial metabolites and specific microbial strains that potentiate as prebiotics, probiotics, or FMT in the clinical settings. All potential interventions are still under investigation or not yet determined. However, the mechanisms that cause dysbiosis post-HSCT, excluding broad-spectrum antibiotics, remain elusive. Because IECs utilize oxygen for energy, the intestinal environment has low oxygen levels (26, 89), resulting in the domination of anaerobic bacteria in the gut microbiota. As shown in an inflammatory bowel disease model, the metabolic change through PPAR-γ signaling in IECs might lead to dysbiosis in GVHD, which still require further investigation (19). The role of SCFAs in cell metabolism suggests that tissue damage can be reduced by directly intervening target cells without substantially affecting the immune system (68, 172). Tolerance mediated by tissue homeostasis reduces immunological damage from T-cells and other cells. In addition, GVHD and GVL can also be separated. Furthermore, the connection between GVL effects and dysbiosis should be explored to identify potential strategies that boost immune reactions.

**Figure 3:**
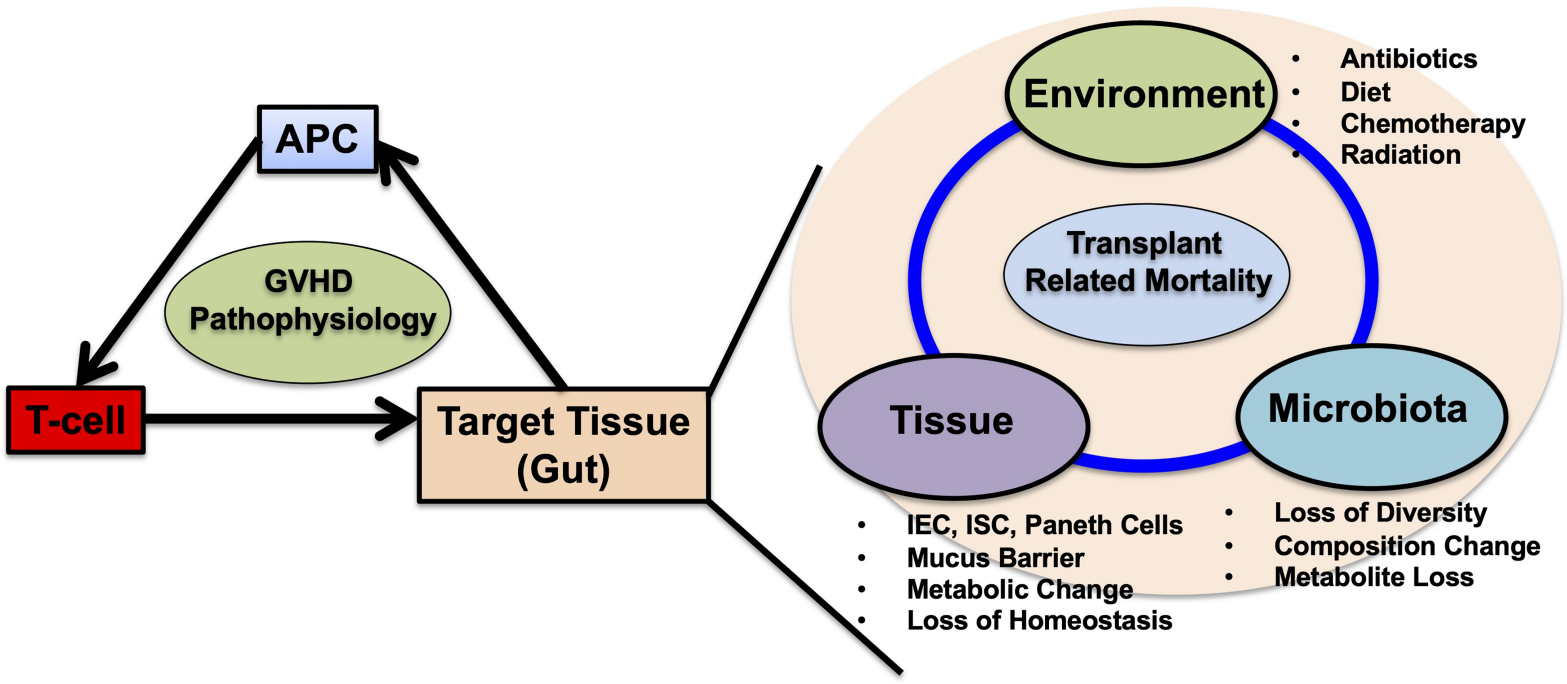
Schematic representation of acute GVHD pathophysiology and the multifactorial interactions in the gut target tissue. Historically, antigen presenting cells (APCs), donor-T cells and target tissues are the main factors in acute GVHD pathophysiology. Among these cells, APCs and T-cells are well studied and targeted for GVHD prevention and treatment. In gut GVHD, microbial dysbiosis are highly associated with transplant related mortality. Microbial dysbiosis is one aspect of multifactorial interactions in the gut during GVHD. In HSCT, conditioning regimens including chemotherapy and irradiation cause damages to intestinal epithelial cells (IECs), intestinal stem cells (ISCs), paneth cells mucus producing goblet cells. Disruption of gut microbiota already exists before HSCT, and prophylactic and systemic antibiotic treatments worsens the microbial dysbiosis with loss of diversity, composition changes along with increase of *Enterococcus*. Dysbiosis depletes microbial derived metabolites changes the IEC metabolism skewing to less tolerant against donor T-cell damages, postpones IEC repairs and mucus barrier restorations. Expansion of Pathogenic bacteria permits blood stream infection thorough injured IECs that necessitate systemic antibiotic administration, leading to further microbial disruptions. These factors including environment, tissue and microbiota falls into the vicious cycle leading to transplant related mortality. The questions remain whether which factor could be prioritized and be targeted for treatments including fecal microbiota transplantation (FMT), prebiotics, probiotics, the attentions of the usages of anti-anaerobic antibiotics and other interventions targeting tissues. Single or combined strategies are warranted to break the vicious cycle and improve HSCT outcomes.

Understanding the precise mechanisms and conditions in the intestinal environment can promote the development of prophylactic/therapeutic strategies targeting single or combined modalities (such as FMT or FMT + butyrate, combinations of metabolites) to reduce immunological reactions originating from the gut (**Figure 3**). Furthermore, investigating the crosstalk between local microbiota and injuries in target organs, such as the lungs, could unveil other strategies to prevent HSCT-related complications (173).

## Author Contributions

The author confirms being the sole contributor of this work and has approved it for publication.

## Funding

This work was supported by JSPS KAKENHI Grant number JP20K22901, JP21H02904, The Kawasaki Foundation of Medical Science and Medical Welfare, The Ryobiteien Memorial Foundation, The MSD Life Science Foundation Public Interest Incorporated Foundation, The Okayama Medical Foundation, The SENSHIN Medical Research Foundation, The Kato Memorial Bioscience Foundation and the NOVARTIS Foundation (Japan) for the Promotion of Science.

## Conflict of Interest

The author declares that the research was conducted in the absence of any commercial or financial relationships that could be construed as a potential conflict of interest.

## Publisher’s Note

All claims expressed in this article are solely those of the authors and do not necessarily represent those of their affiliated organizations, or those of the publisher, the editors and the reviewers. Any product that may be evaluated in this article, or claim that may be made by its manufacturer, is not guaranteed or endorsed by the publisher.
